# Genome Sequences of Three *Microbacterium* Phages Isolated from Flowers

**DOI:** 10.1128/MRA.01468-18

**Published:** 2019-01-03

**Authors:** Kerry S. Iles, Kira M. Zack, Alyssa J. Betsko, Rebecca A. Garlena, Daniel A. Russell, Andrea Fetters, Deborah Jacobs-Sera, Tia-Lynn Ashman, Graham F. Hatfull

**Affiliations:** a Department of Biological Sciences, University of Pittsburgh, Pittsburgh, Pennsylvania, USA; Indiana University, Bloomington

## Abstract

Bacteriophages Balsa, Golden, and Lucky3 are cluster EA phages isolated from flowers and infect Microbacterium foliorum NRRL B-24224. The genomes of Golden and Lucky3 (subcluster EA1) are closely related, whereas Balsa (subcluster EA4) is a more distant relative.

## ANNOUNCEMENT

Numerous bacteriophages infecting actinobacterial hosts have been isolated from environmental samples, such as soil and compost (1–3). The presence of actinobacteria on internal and external structures of flowers suggests that phages of these hosts might also be present (4). We report here the genome sequences of phages Balsa, Golden, and Lucky3, which were isolated from flowers of Impatiens pallida (Balsaminaceae), Solidago canadensis (Asteraceae), and Trifolium repens (Fabaceae), respectively, collected in Pittsburgh, PA, using Microbacterium foliorum NRRL B-24224 as a bacterial host. In brief, flowers were washed in peptone-yeast extract-calcium (PYCa) broth, phage growth was enriched by incubation with host bacteria in PYCa broth at 30°C, and plaques were identified following plating on PYCa agar. Phage Balsa forms turbid plaques, whereas Golden and Lucky3 form clear plaques of various sizes.

Phage DNA was isolated from phage lysates using the Wizard DNA extraction kit (Promega), and then sequencing libraries were prepared using an NEB Ultra II FS kit and run on an Illumina MiSeq platform, yielding at least 100,000 single-end 150-base reads for each genome. Reads were assembled using Newbler 2.9, with default settings, and in each case yielded a single phage contig (average coverages for Golden, Lucky3, and Balsa, 1,675-, 3,441-, and 1,992-fold, respectively) which was evaluated with Consed 29. No evidence was found for defined genomic termini (5); therefore, the start of the terminase small subunit was chosen as the genome coordinate terminus. Balsa has a 41,862-bp genome with 63.4% G+C content, while Golden and Lucky3 each have 39,640-bp genomes with 64.1% G+C content. The G+C content of the host (68.7%; accession number NZ_CP031425) is somewhat higher than that of the three phages (our unpublished data). All three phages belong to cluster EA and share at least 35% average gene content (2). Golden and Lucky3 differ by only three single-base substitutions and were grouped in subcluster EA4. Balsa is similar to a large number of phages grouped in subcluster EA1.

The three genomes were annotated using a previously described genome annotation pipeline (6), together with Glimmer (7), GeneMark (8), BLASTP (9), HHPred (10), and Phamerator (11). Golden and Lucky3 both have 58 predicted protein-coding genes, and Balsa has 62 protein-coding genes. The genome leftmost halves are transcribed rightwards and code for virion structure and assembly, followed by a lysis cassette. With the exception of the rightmost two genes of each genome, the rightmost genome halves are transcribed leftwards and contain genes coding for DNA metabolism functions, including DNA polymerase I, helicase, thymidylate synthase, exonuclease, and a RecA-like recombinase. Although Balsa forms turbid plaques, we did not identify genes typically found in temperate phages, such as those for a repressor, integrase, or partitioning functions.

All three phages share common virion structure and assembly genes, including a putative HK97-fold capsid subunit and a tape measure protein gene. The lysis cassettes contain putative holin and endolysin genes, although the Balsa endolysin (gp24), with d-Ala-d-Ala carboxypeptidase and transglycosylase domains, differs from those in Golden and Lucky3 (gp24) that have peptidoglycan binding and amidase domains. Balsa contains several genes near its right end that are absent in subcluster EA4 phages. Only 11 genes found in these phages have homologues in phages outside cluster EA.

### Data availability.

The GenBank and Sequence Read Archive accession numbers are MG839030 and SRR7769840 for Balsa, MG925343 and SRR7769839 for Golden, and MG925347 and SRR7769841 for Lucky3, respectively.
